# Unmasking Protein Phosphatase 2A Regulatory Subunit B as a Crucial Factor in the Progression of Dilated Cardiomyopathy

**DOI:** 10.3390/biomedicines12081887

**Published:** 2024-08-19

**Authors:** Fang Lin, Xiaoting Liang, Yilei Meng, Yuping Zhu, Chenyu Li, Xiaohui Zhou, Sangyu Hu, Na Yi, Qin Lin, Siyu He, Yizhuo Sun, Jie Sheng, Huimin Fan, Li Li, Luying Peng

**Affiliations:** 1 State Key Laboratory of Cardiology and Medical Innovation Center, Shanghai East Hospital, Tongji University School of Medicine, Tongji University, Shanghai 200120, China; 1911242@tongji.edu.cn (F.L.);; 2 Laboratory of Molecular Genetics and Stem Cell Differentiation, Tongji University School of Medicine, Tongji University, Shanghai 200120, China; 3 Department of Cell and Genetics, Tongji University School of Medicine, Tongji University, Shanghai 200120, China; 4 Research Center for Translational Medicine, Shanghai East Hospital, Tongji University School of Medicine, Tongji University, Shanghai 200120, China; 5 Shanghai Heart Failure Research Center, Shanghai East Hospital, Tongji University School of Medicine, Tongji University, Shanghai 200120, China; 6 Institute for Regenerative Medicine, Shanghai East Hospital, Tongji University School of Life Sciences and Technology, Tongji University, Shanghai 200120, China

**Keywords:** *Ppp2r5d*, dilated cardiomyopathy, STAT3, phosphorylation, mitochondria

## Abstract

Dilated cardiomyopathy (DCM) is one of the major causes of heart failure. Although significant progress has been made in elucidating the underlying mechanisms, further investigation is required for clarifying molecular diagnostic and therapeutic targets. In this study, we found that the mRNA level of protein phosphatase 2 regulatory subunit B’ delta (*Ppp2r5d*) was altered in the peripheral blood plasma of DCM patients. Knockdown of *Ppp2r5d* in murine cardiomyocytes increased the intracellular levels of reactive oxygen species (ROS) and inhibited adenosine triphosphate (ATP) synthesis. In vivo knockdown of *Ppp2r5d* in an isoproterenol (ISO)-induced DCM mouse model aggravated the pathogenesis and ultimately led to heart failure. Mechanistically, *Ppp2r5d*-deficient cardiomyocytes showed an increase in phosphorylation of STAT3 at Y705 and a decrease in phosphorylation of STAT3 at S727. The elevated levels of phosphorylation at Y705 in STAT3 triggered the upregulation of interleukin 6 (IL6) expression. Moreover, the decreased phosphorylation at S727 in STAT3 disrupted mitochondrial electron transport chain function and dysregulated ATP synthesis and ROS levels. These results hereby reveal a novel role for *Ppp2r5d* in modulating STAT3 pathway in DCM, suggesting it as a potential target for the therapy of the disease.

## 1. Introduction

Dilated cardiomyopathy (DCM) is characterized by dilated ventricles and impaired myocardial contractile capacity, generally leading to cardiac dysfunction, arrhythmias, and finally heart failure. Cardiomyocytes from DCM patients exhibit a progressive increase in apoptosis, which results in compensatory hypertrophy of the healthy remaining cardiomyocytes. While many factors, including genetic mutations, autoimmune diseases, inflammation and infection, toxins, excessive alcohol consumption, and chemotherapeutic drugs, have been observed to be associated with the occurrence of DCM, still others have yet to be identified [1].

Our previous study identified an altered mRNA level of protein phosphatase 2 regulatory subunit B’ delta (*Ppp2r5d*) in the plasma of DCM patients as compared to that of ischemic cardiomyopathy (ICM) patients and healthy individuals (GEO: GSE138678) [2]. The abnormal alteration of *Ppp2r5d* was further confirmed in DCM mice, which suggests that *Ppp2r5d* may serve as a potential diagnostic biomarker for DCM. Protein phosphatase 2A (PP2A) is one of the four major Ser/Thr phosphatases that negatively control cell growth and division. *Ppp2r5d*, encoding B56δ (a delta isoform of the regulatory subunit B56 subfamily of PP2A) has been shown to be involved in neurodevelopment and tumorigenesis [3], and its mutant leads to mental retardation and neurodevelopmental disorder [4,5,6]. On the other hand, isoproterenol (ISO)-stimulated cardiomyocytes can induce the phosphorylation of subunit B56δ at the Ser573 site, thereby enhancing PP2A activity. Additionally, cTnI and cMyBP-C are phosphorylated by PKA following ISO stimulation. Inhibiting PP2A activity can increase the levels of phosphorylation at the intracellular cTnI Ser22/23 and cMyBP-C Ser282 sites [7]. The double knockout of *Ppp2r5d* and *Ppp2r5c* arrest embryonic development at around 12 days, accompanied by the apoptosis of heart tissue and by abnormal cardiac vascular development in mice [8]. All these studies indicate that *Ppp2r5d* plays a crucial role in the development of cardiac disease. Here, we elucidate that *Ppp2r5d* knockdown in mouse cardiomyocytes enhances the inflammatory response and aggravates mitochondrial energy metabolism, thereby accelerating DCM progression. Our findings demonstrate that *Ppp2r5d* indeed modulates the pathogenesis of DCM.

## 2. Materials and Methods

### 2.1. Animals

Four-to-six-week-old male C57BL/6J mice were purchased from Vital River (Beijing, China). All animal experiments were approved by the Animal Care and Use Committee of Tongji University (Permit Number: TJAA00222101), and the procedures followed the National Research Council’s Guide for the care and use of laboratory animals. A DCM mouse model was induced by ISO treatment. Briefly, mini-osmotic pumps (2002W, RWD Life Science, Shenzhen, China) containing saline or ISO (I5627, Sigma-Aldrich, St. Louis, MO, USA; 60 mg/kg/day) were implanted subcutaneously for two weeks.

The myocardial-specific *Ppp2r5d* knockdown was achieved through delivery of adeno-associated virus 9 (AAV9) carrying shRNA against *Ppp2r5d* (AAV9-cTnT-shRNA-*Ppp2r5d*-GFP) driven with a cTnT promoter via tail vain injection (5 × 10^11^ viral particles per animal in 100 μL, Hanheng Biotechnology, Shanghai, China). Mice receiving an equivalent amount of AAV9-cTnT-shRNA-NC (5 × 10^11^ viral particles per animal in 100 μL, Hanheng Biotechnology, Shanghai, China) served as the control group. At week 3, the mice were subcutaneously implanted with a mini-osmotic pump containing ISO to induce DCM as previously described. After an additional eight weeks, echocardiography was performed and cardiac tissues were collected for histological and biochemical analysis.

### 2.2. Cell Culture

The mouse cardiomyocytes (MCMs) were purchased from the ScienCell research laboratories. The passage number in the experiment was between 10 and 20. Cells were cultured in Dulbecco’s modified Eagle’s medium (DMEM, HyClone, Logan, UT, USA) supplemented with 2% fetal bovine serum (FBS) at 37 °C in a humidified 5% CO_2_–95% air atmosphere. Primary neonatal mouse cardiomyocytes cells were cultured in DMEM (HyClone, Logan, UT, USA) supplemented with 10% fetal bovine serum (FBS) at 37 °C in a humidified 5% CO_2_–95% air atmosphere.

The primary neonatal mouse cardiomyocytes were isolated according to the manufacturer’s instructions (AC-1002016, Applied Cell, Shanghai, China). The ventricles of the hearts of P1 or P2 neonatal mice were rinsed with wash buffer and then minced into 1–3 mm^3^ tissue fragments. Initial digestion with mouse cell dissociation solution I was carried out overnight at 4 °C, followed by repeated treatment with dissociation solution II at 37 °C until the cells were fully dissociated. The resulting cell suspensions, comprising both myocytes and non-myocytes, were pooled and then subjected to purification through differential adhesion. The purified cardiomyocytes were subsequently transfected with siRNA for further experiments.

### 2.3. RNA Interference

To knockdown *Ppp2r5d*, MCMs were transfected with either siRNA against *Ppp2r5d* or negative control (NC) siRNA using Lipofectamine™ RNAiMAX transfection reagent (13778030, Thermo Fisher, Waltham, MA, USA) according to the manufacturer’s instructions. The siRNA sequence was as follows: siRNA-*Ppp2r5d*: 5′-AGATCAACCACATCTTCTA-3′. The efficiency of knockdown was assessed 48 h post-transfection through real-time quantitative (RT-qPCR) and Western blot analysis prior to subsequent experiments.

### 2.4. Microarray Assay

The Agilent Sure Print G3 Mouse Gene Expression v2 Microarrays (8 × 60 K, DesignID: 074809) were used, and data analysis was conducted by OE Biotechnology Co., Ltd. (Shanghai, China).

Total RNA was quantified with the NanoDrop ND-2000 (Thermo Fisher Scientific, Waltham, MA, USA), and RNA integrity was assessed with the Agilent Bioanalyzer 2100 (Agilent Technologies, Santa Clara, CA, USA). Sample labelling, microarray hybridization, and washing steps were performed according to the manufacturer’s protocols. Briefly, the total RNA was reverse transcribed to double-strand cDNA, and then synthesized cRNA was labelled with *Cyanine* 3-CTP and hybridized onto the microarray. After washing, the arrays were scanned by the Agilent Scanner G2505C (Agilent Technologies). Raw data were acquired using Feature Extraction software (version 10.7.1.1, Agilent Technologies), and basic analysis was performed using Genespring software (version 14.8, Agilent Technologies). The statistically significant differentially expressed genes were then identified (fold ≥ 2.0 and *p* value ≤ 0.05 by *t*-test). The Gene Ontology (GO) and Kyoto Encyclopedia of Genes and Genomes (KEGG) enrichment analyses of the differentially expressed genes were conducted using SciPy (version 1.8.0) based on hypergeometric distributions.

### 2.5. ATP Concentration Assay

MCMs were initially transfected with siRNA-*Ppp2r5d* and control siRNA-NC, followed by ISO treatment 48 h post-transfection. Cells were harvested 18 h later for in vitro adenosine triphosphate (ATP) concentration analysis using an ATP assay kit (S0027, Beyotime, Nantong, China) according to the manufacturer’s instructions.

### 2.6. Tissue Histopathology

After the echocardiography assessment, the heart tissues were fixed, embedded in paraffin, and sectioned. Masson’s trichrome staining was used to assess fibrosis in the heart sections. Wheat germ agglutinin (WGA) (L4985, Sigma-Aldrich, St. Louis, MO, USA) was utilized for staining cardiac sections to measure the area of cardiomyocytes according to the manufacturer’s instructions. Images were captured by fluorescence microscopy (Leica Microsystems, Wetzlar, Germany).

### 2.7. Real-Time Quantitative PCR

RNA was extracted with TRNzol (DP424, TIANGEN, Beijing, China). The cDNA was synthesized using a PrimerScriptTM RT Master Mix (RR036, Takara, Beijing, China). Quantitative reverse transcription polymerase chain reaction (RT-qPCR) analysis was conducted with SYBRTM green master mix according to the manufacturer’s protocol (4385617, Thermo Fisher Scientific, Waltham, MA, USA). The expression of target genes was normalized to the expression level of control using the 2^−ΔΔCt^ cycle threshold method. *β-actin* and *Vdac1* were utilized as endogenous controls separately. The sequence of primers is listed in Table S1.

### 2.8. Enzyme-Linked Immunosorbent Assay (ELISA)

The supernatant of cell cultures was collected and centrifuged to remove cell debris after 3 days of transfection. The interleukin 6 (IL6) concentration was determined with a commercial ELISA kit according to the manufacturer’s instructions (EK206/3-96, MultiScience, Shanghai, China).

### 2.9. Mitochondrial Respiration Assay

The Seahorse XFe96 assay (Agilent Technologies, Santa Clara, CA, USA) was used to quantify the oxygen consumption rate (OCR) of MCMs. MCMs were transfected with siRNA-*Ppp2r5d* and siRNA-NC. For this aim, 1.5 × 10^4^ of cells were seeded in customized Seahorse 96-well plates and treated with ISO (50 μM) for 18 h. Then, the medium was replaced with DMEM (Seahorse Bioscience, Billerica, MA, USA), supplemented with 1 mM pyruvate, 2 mM glutamine, and 10 mM D-glucose. The OCR was determined using the Seahorse Bioscience XF96 Extracellular Flux Analyzer (Seahorse Bioscience, Billerica, MA, USA). Measurements were made as the cells were incubated sequentially under four conditions: 1. basal levels were measured in the absence of additives; 2. oligomycin (1.5 μM) was added to reversibly inhibit oxidative phosphorylation (OXPHOS) and thus ATP synthase to measure glycolytic ATP synthesis; 3. FCCP (1 μM), a mitochondrial uncoupler, was added to disrupt OXPHOS-dependent ATP generation; 4. antimycin A (10 μM), a complex I inhibitor and mitochondrial poison, was added to stop OXPHOS-dependent ATP production. The ATP levels under these four different conditions were calculated using Seahorse Wave Controller Software (version 2.4), with OCR normalized to the number of cells in each well.

### 2.10. Assay for Intracellular Reactive Oxygen Species

To assess intracellular ROS level, MCM cells were transfected with siRNA-*Ppp2r5d* and siRNA-NC. After two days, the MCMs were switched to glucose-free medium overnight, following stimulation with H_2_O_2_ (400 μM) for 1.5 h. MCMs from the control, siRNA-NC, and siRNA-*Ppp2r5d* groups were treated with 10 μM of DCFH-DA dye for 1 h at 37 °C, followed by three washes with serum-free DMEM. Subsequently, the DCF fluorescence was promptly measured using flow cytometry (BD LSRII flow cytometer, excitation/emission: 488/525 nm) according to the manufacturer’s instructions (S0033, Beyotime, Nantong, China).

### 2.11. Assay for MitoTracker Green

To measure the intracellular mitochondria, MitoTracker green dye was used according to the manufacturer’s instructions (40742ES50, Yeason, Xi’an, China). In brief, MCM cells from the control, siRNA-NC, and siRNA-*Ppp2r5d* groups were harvested and incubated with MitoTracker green (100 nM) for 30 min at 37 °C, following washing with PBS. Fluorescence intensity was immediately measured with flow cytometry (BD LSRII flow cytometer; excitation/emission: 490/523 nm).

### 2.12. Mitochondria Isolation and Assay for ETC Activity

Mitochondria from MCM were isolated using a mitochondrial isolation kit (MM-038, Invent Biotechnologies, Plymouth, MN, USA), and then the activity of electron transport chain (ETC) complex I in the purified mitochondria was determined according to the manufacturer’s protocol (BC0515, Solarbio, Beijing, China). Briefly, the activity was quantified by measuring the oxidation of NADH at 340 nm according to the manufacturer’s instructions. Protein content was determined by the bicinchoninic acid (BCA) method, and values are expressed as activities in nanomoles substrate consumed per minute per milligram of protein.

### 2.13. Echocardiography

Cardiac performance was evaluated at 6 weeks post-ISO-infusion by echocardiography. Mice were anaesthetized with isoflurane (2% isoflurane for induction and 1% for maintenance). Left ventricular images were obtained in the parasternal long-axis view using a Vevo 2100 Imaging System (Fujifilm Visual Sonics, Toronto, ON, Canada) equipped with a 30 Mhz MicroScan transducer (model MS-400). As previously described, LV mass and functional parameters were calculated, such as ejection fraction and fractional shortening [9].

### 2.14. Transmission Electron Microscopy (TEM) Analysis

Heart tissues were rapidly fixed in 2.5% glutaraldehyde for 2 h and then trimmed to 1~3 mm^3^ in size. After three washes with 0.1 M of phosphate buffer, the tissues were fixed with 1% osmic acid at 4 °C for 2 h, followed by rinsing and gradient dehydration through graded ethyl alcohols. Subsequently, they were embedded in Epon-Araldite resin, and the resulting clumps were polymerized in copper. Semi-thin sections were initially utilized for localization, while ultrathin sections were subsequently employed for microstructural analysis. After counterstaining was completed with uranyl acetate and lead citrate, the specimens were examined under an HT7800 transmission electron microscope. The images were finally analyzed using ImageJ to quantify the mitochondria surface area (µm^2^) and perimeter (µm).

### 2.15. Western Blot

The total protein was extracted from MCMs with RIPA lysis buffer containing phosphatase and protease inhibitors (P0013B, Beyotime, Nantong, China), and the protein concentrations were quantified using the BCA Protein Assay Kit (P0010, Beyotime, Nantong, China). The protein samples were separated by SDS-PAGE and transferred to the hydrophobic PVDF membrane. Blotted membranes were incubated with primary antibodies at 4 °C overnight and then incubated with specific secondary antibodies at room temperature for 1 h. The targeted proteins were visualized by enhanced chemiluminescence (34094, ThermoFisher, Waltham, MA, USA) with a Tanon 1600 apparatus (Tanon, Shanghai, China) according to the manufacturer’s instructions. The optical intensities of the blotted bands were quantified using ImageJ software (version 1.4.3.67). The following primary antibodies were used: anti-Phospho-STAT3 (Y705) (9145, CST, New York, NY, USA); anti-Phospho-STAT3 (S727) (9136, CST, New York, NY, USA); anti-STAT3 (4904; CST, New York, NY, USA); anti-*Ppp2r5d* (B56δ) (12068-1-AP, Proteintech, Wuhan, China); anti-GADPH-HRP (Pab001H, ESscience, China); anti-VDAC1/2 (10866-1-AP, Proteintech, Wuhan, China); and anti-Total OXPHOS Rodent WB antibody Cocktail (ab110413, Abcam, Cambridge, UK).

### 2.16. Statistical Analysis

All data are presented as the mean ± SD. Statistical analyses were performed using GraphPad Prism 5.04 software. Comparisons between two groups were analyzed by the unpaired Student’s *t*-test, and comparisons between more than two groups were analyzed by one-way ANOVA followed by the Bonferroni test. A value of *p* < 0.05 was considered statistically significant.

## 3. Results

### 3.1. Ppp2r5d Was Associated with DCM Pathogenesis

**Figure 1:**
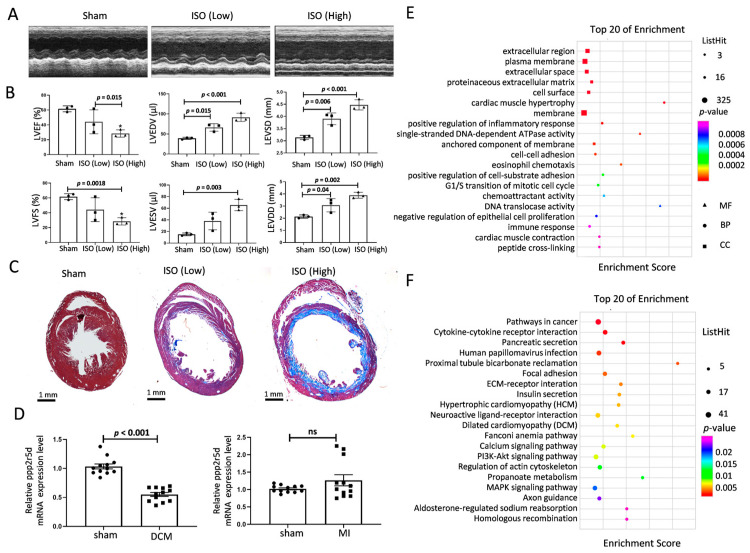
Characterization of the ISO-induced DCM mouse model. (**A**) Representative images of DCM mode echocardiography in each group. ISO (Low): 30 mg/kg/day; ISO (high): 80 mg/kg/day. (**B**) Quantitative analysis of the echo parameters of the left ventricle of each group. LVEF: left ventricular ejection fraction, LVFS: left ventricular fraction shortening, LVEDV: LV end-diastolic volume, LVESV: LV end-systolic volume, LEVDD: left ventricular end-diastolic dimension, LEVSD: left ventricular end-systolic dimension (*n* = 3). (**C**) Masson stain of the short-axis section of hearts. Scale bar: 1 mm. (**D**) The expression of *Ppp2r5d* mRNA in the cardiac ventricles of DCM mice and MI mice was compared with that of sham mice using RT-qPCR (*n* = 4). (**E**) GO enrichment analysis of DEGs in *Ppp2r5d* knockdown with MCMs vs. control MCMs. (**F**) KEGG pathways of DEGs in *Ppp2r5d* knockdown with MCMs vs. control MCMs. Data are presented as the mean ± SD with *p* values indicated. * *p* < 0.05.

Given that activation of β-adrenergic receptors (β-AR) by ISO can induce progressive left ventricular dilatation, cardiac dysfunction, and fibrosis [10,11], a subcutaneous osmotic mini-pump was used here to apply ISO for 2 weeks to induce DCM in mice. The results revealed typical cardiac dysfunction, including increased left ventricular end-diastolic dimension (LVEDD) and left ventricular end-systolic dimension (LEVSD), as well as decreased left ventricular ejection fraction (LVEF) and left ventricular fraction shortening (LVFS) after 8 weeks of ISO infusion (Figure 1A,B). Additionally, an exacerbation of dilated left ventricular and myocardial fibrosis was also detected in the ISO-induced mice (Figure 1C), indicating the successful establishment of the DCM mice model.

Since *Ppp2r5d* mRNA was altered in the peripheral plasma of DCM patients (GEO: GSE138678), we then checked the status of *Ppp2r5d* in mice cardiac tissues. There was a significant decrease in the level of *Ppp2r5d* in the ventricular tissues of the mouse model, and there was no difference in the myocardial infarction (MI) mouse model (Figure 1D). To further confirm this change was associated with the pathogenesis of DCM, we knocked down *Ppp2r5d* in MCMs using siRNA-*Ppp2r5d* and then performed gene expression microarray assay to identify the differentially expressed genes (DEGs). Further enrichment analysis showed that the DEGs are highly involved in cardiac muscle hypertrophy and cardiac muscle contraction in Gene Ontology (GO) (Figure 1E) and are highly associated with hypertrophic cardiomyopathy (HCM) and DCM pathway in Kyoto Encyclopedia of Genes and Genomes (KEGG) (Figure 1F). Collectively, these results indicate that *Ppp2r5d* is indeed involved in DCM pathogenesis.

### 3.2. Ppp2r5d Knockdown Increased Oxidative Stress and Mitochondrial Dysfunction in MCMs

**Figure 2:**
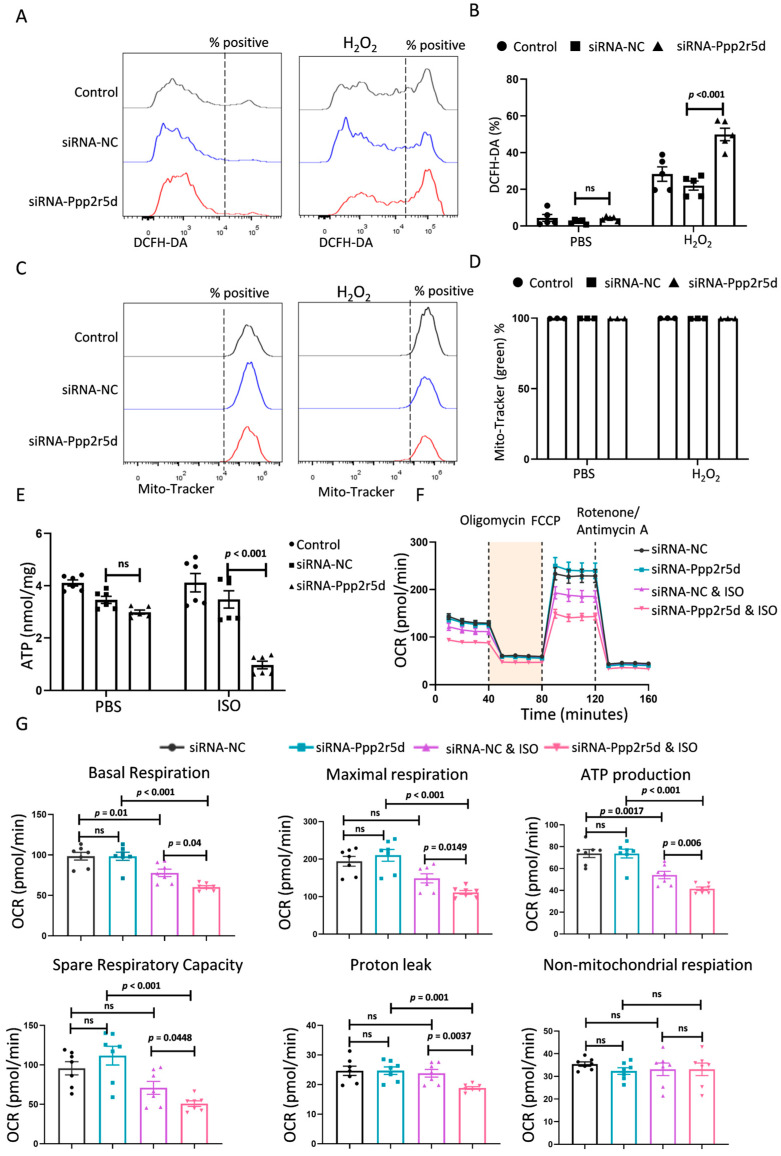
The effects of *Ppp2r5d*-knockdown MCMs on mitochondrial dysfunctions. (**A**) Representative histogram plots show the ROS levels in MCMs after treatment. (**B**) Quantification of DCFH-DA staining (Mean ± SD, *n* = 5). (**C**) Representative histogram plots show the mitochondria levels in MCMs after treatment. (**D**) Quantification of MitoTracker green staining (Mean ± SD, *n* = 3). (**E**) ATP production in MCMs was measured with an ATP kit (Mean ± SD, *n* = 6). (**F**) Mitochondrial respiration in MCMs was measured with the Seahorse assay. MCMs were sequentially incubated with oligomycin (1.5 μM), FCCP (1 μM), and antimycin (10 μM). (**G**) The mitochondrial functional parameters from F (basal respiration, maximal respiration, ATP production, spare respiratory capacity, non-mitochondrial respiration, and proton leak). *n* = 7. Data are presented as the mean ± SD with *p* values indicated.

Oxidative stress characterized by the accumulation of reactive oxygen species (ROS) mediates the development of DCM [12,13]. Next, we checked the ROS status in *Ppp2r5d*-deficient cardiomyocytes after the hydrogen peroxide (H_2_O_2_) challenge. As shown in Figure 2A,B, although H_2_O_2_ treatment could increase ROS level in MCMs, a more significant increase was detected in *Ppp2r5d*-deficient MCMs, which then led to obvious apoptosis of MCMs that was confirmed again with a significant increase in the level of cleaved caspase 3 (Figure S1). Intracellular ROS generation was also detected by the fluorescent probe dihydroethidium (DHE). After ISO stimulation, the DHE staining indicated a significant increase between the *Ppp2r5d*-deficient MCMs and the control MCMs (Figure S2). Collectively, *Ppp2r5d* knockdown enhanced ROS generation in MCMs after ISO and H_2_O_2_ stimulation.

Mitochondria are major sources of intracellular reactive oxygen species, and excessive ROS production may induce mitochondrial dysfunction [14]. *Ppp2r5d* deficiency has no significant influence on mitochondrial mass (Figure 2C,D), but whether it has an effect on mitochondrial energy metabolism and function needs further clarification. We found that knockdown of *Ppp2r5d* modestly reduced basal ATP production in MCMs, and significant inhibition was detected in the presence of ISO (Figure 2E). ATP is primarily produced in mitochondria through the oxidative phosphorylation process, suggesting that *Ppp2r5d* deficiency in MCMs may attenuate mitochondrial biogenesis and/or mitochondrial respiration [15]. Bioenergetic profiles of MCMs were then determined by the Seahorse XF cell Mito stress test with the oxygen consumption rate (OCR) being calculated as an indicator of mitochondrial respiration. The OCR assay showed that only downregulation of *Ppp2r5d* with stimulation of ISO induced a significant decrease in basal respiration, maximal respiration, spare respiratory capacity, proton leak, and ATP production (Figure 2F,G). These results indicate that *Ppp2r5d* deficiency aggravates the ISO-induced mitochondrial dysfunction in vitro.

### 3.3. Ppp2r5d Knockdown Aggravated Cardiac Dilation and HF

**Figure 3:**
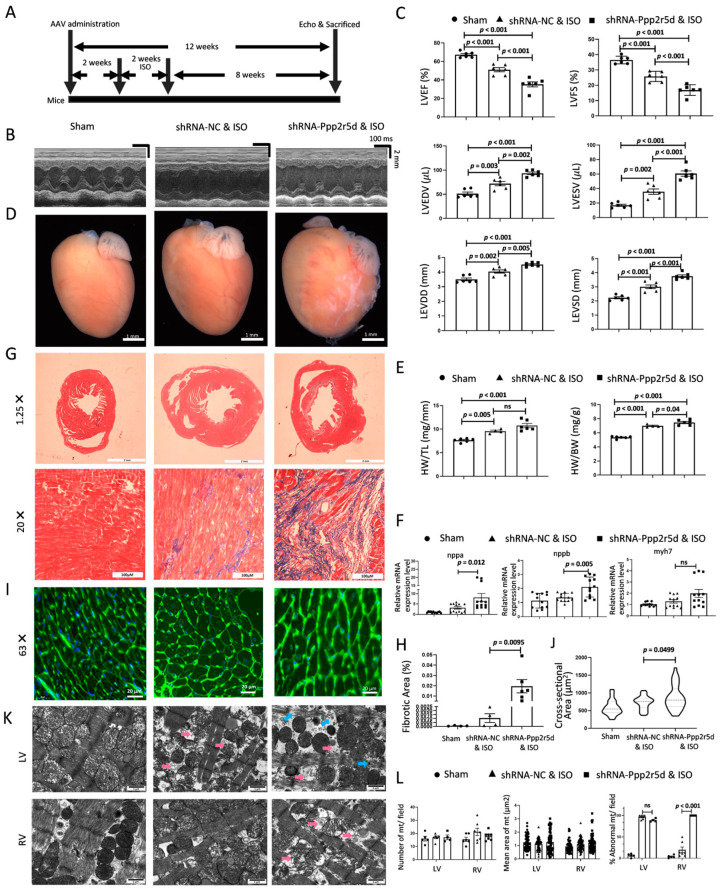
The effects of cardiomyocyte-specific *Ppp2r5d* knockdown on the pathogenesis of DCM and heart failure (HF) in vivo. (**A**) Workflow of the animal experiments. (**B**) Representative echocardiographic images at 8 weeks after removal of the osmotic mini-pump are shown. (**C**) Quantitation of the left ventricles’ parameters in each group. LVEF: left ventricular ejection fraction; LVFS: left ventricular fraction shortening; LVEDV: LV end-diastolic volume; LVESV: LV end-systolic volume; LEVDD: left ventricular end-diastolic dimension; LEVSD: left ventricular end-systolic dimension. *n* = 6. (**D**) Representative anatomical images of the hearts. Scale bar: 1 mm. (**E**) Heart weight/body weight ratio and heart weight/tibia length ratio in each group of mice. *n* = 6. (**F**) Relative mRNA expression of *Nppa*, *Nppb*, and *Myh7* in the ventricles of sham mice, AAV-cTnT-shRNA-NC & ISO mice, and AAV-cTnT-shRNA-*Ppp2r5d* & ISO mice. *n* = 6. (**G**) Masson stain of the collagen deposition of the lateral cross-section of hearts (Mag 1.25× and 20×). (**H**) Quantification of fibrosis area percentage. (**I**) WGA stain of the lateral cross-section of hearts (Mag 63×). (**J**) Quantification of cell area (μm^2^). (**K**) Representative transmission electron microscopy (TEM) images of the left ventricular (LV) and right ventricular (RV) section ultrastructure from sham mice (left), AAV-cTnT-shRNA-NC & ISO mice (middle), and AAV-cTnT-shRNA- *Ppp2r5d* & ISO mice (right). Magnification: 5000×; scale bar: 1 μm. Red arrows: vacuolated mitochondria; blue arrows: broken myocardial muscle fibers. (**L**) Quantification of mitochondria-related parameters from the panel: the number of mitochondria per field, the mean area of the mitochondria in each image, and the percentage of the abnormal mitochondria per field, respectively. Data are presented as the mean ± SD with *p* values indicated.

To further evaluate the potential role of *Ppp2r5d* in DCM progression in vivo, adeno-associated virus (AAV) serotype 9 was used to mediate cardiomyocyte-specific knockdown of *Ppp2r5d*. Western blot revealed that B56δ could be expressed in multiple murine tissues, but only downregulated in the cardiac tissue after AAV-cTnT-shRNA-*Ppp2r5d* injection. There was no noticeable difference in the cardiac phenotype between the AAV-cTnT-shRNA-*Ppp2r5d* and AAV-cTnT-shRNA-NC groups (Figure S3). A flowchart of the animal experiment is shown in Figure 3A. Cardiac function was assessed using echocardiography at eight weeks after ISO infusion. Compared to the sham group, the ISO-treated mice exhibited an increase in left ventricular diameter and volume, accompanied by a reduction in left ventricular fractional shortening (FS) and ejection fraction (EF). Notably, the differences were more prominent in the AAV-cTnT-shRNA-*Ppp2r5d* & ISO group than in the control AAV-cTnT-shRNA-NC & ISO group (Figure 3B,C). Consistent with the echocardiography results, there was a notable increase in heart size and in both the heart weight-to-body-weight ratio (HW/BW) and heart weight-to-tibia-length ratio (HW/TL) in AAV-cTnT-shRNA-*Ppp2r5d* & ISO mice (Figure 3D,E). In addition, ISO led to an increase in mRNA level of classical-cardiac-hypertrophy- and stress-related genes in the left ventricle, particularly under *Ppp2r5d*-knockdown conditions, including atrial natriuretic peptide (Nppa), brain natriuretic peptide (Nppb), and myosin heavy chain (Myh7) (Figure 3F). Masson staining of cardiac tissue revealed that *Ppp2r5d* deficiency aggravated ISO-induced cardiac fibrosis (Figure 3G,H). Moreover, the increased cardiomyocyte size suggested that *Ppp2r5d* deficiency also aggravated ISO-induced cardiomyocyte hypertrophy (Figure 3I,J).

Since *Ppp2r5d* deficiency was involved in ISO-induced mitochondrial dysfunction in vitro (Figure 2F), we proceeded to investigate the potential impact of *Ppp2r5d* inhibition on mitochondria morphology in vivo. The ultrastructure analysis revealed that the mitochondria of the left ventricular tissue in the AAV-cTnT-shRNA-NC & ISO mice exhibited various abnormalities, including swelling, vacuolization, membrane rupture, and reduced cristae, while no significant alterations were observed in the right ventricular tissue. However, the *Ppp2r5d*-deficient DCM mice exhibited mitochondrial abnormalities in both left and right ventricles (Figure 3K,L). These results suggest that mitochondrial dysfunction induced by *Ppp2r5d* downregulation in the DCM model truly contributes to cardiac dilation and heart failure in vivo.

### 3.4. Ppp2r5d Knockdown Impaired ETC Complex Activity

**Figure 4:**
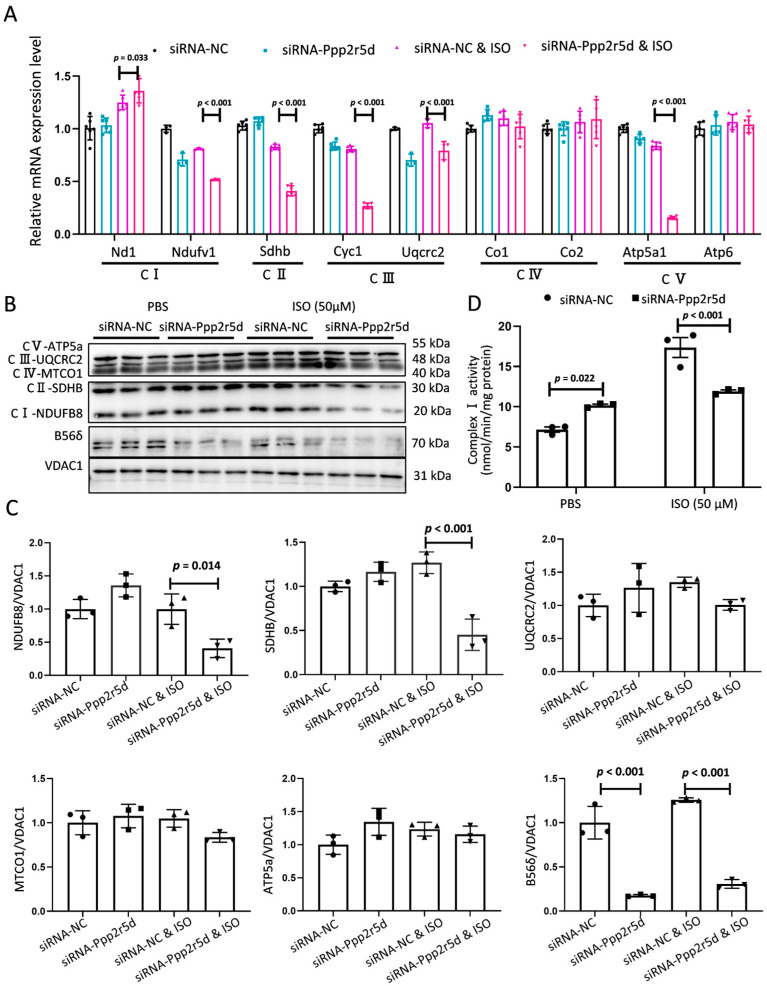
Knockdown of *Ppp2r5d* impaired the activity of the mitochondrial respiratory chain complex in MCMs. (**A**) Relative mRNA expression of the mitochondrial genes in each group. (**B**) Protein expression of mitochondrial complexes assessed by Western blot in each group. Experiments were repeated three independent times. (**C**) The relative band intensity of NDUFB8, SDHB, UQCRC2, MTCO1, ATP5a, and B56δ were analyzed by Image J software (version 1.4.3.67) and normalized to total VDAC1. (**D**) Mitochondria were isolated from MCMs, and complex I activity was measured. The enzymatic activity is presented as milliunits (nmol/min) per milligram (mg) of mitochondrial protein. Data are presented as the mean ± SD with *p* values indicated.

Electron transport via the mitochondrial electron transport chain (ETC) complex I, II, III, and IV generates an electrochemical proton gradient, which is then utilized by ATP synthase to produce ATP [16]. Based on the observation that *Ppp2r5d* deficiency altered ROS generation (Figure 2A) and ATP production (Figure 2E) in MCMs under stimulation, we hypothesized that impairments of ETC capacity may be responsible for mitochondrial dysfunction in MCMs. Therefore, we proceeded to examine the expression patterns of genes associated with the ETC complex involved in this process. After ISO treatment, inhibition of *Ppp2r5d* triggered a significant transcriptional downregulation of *Ndufv1, Sdhb, Cyc1, Uqcrc2, and Atp5a1* (Figure 4A). Some protein levels, including NDUFB8 and SDHB, were accordingly decreased (Figure 4B,C). Moreover, the activity of ETC complex I was also significantly inhibited (Figure 4D). Taken together, these results demonstrate that *Ppp2r5d* knockdown impairs the expression of the ETC complex and exacerbates ISO-induced cardiac dysfunction.

### 3.5. Ppp2r5d Knockdown Induced Bidirectional Phosphorylation of STAT3 in the Hypertrophy Model

**Figure 5:**
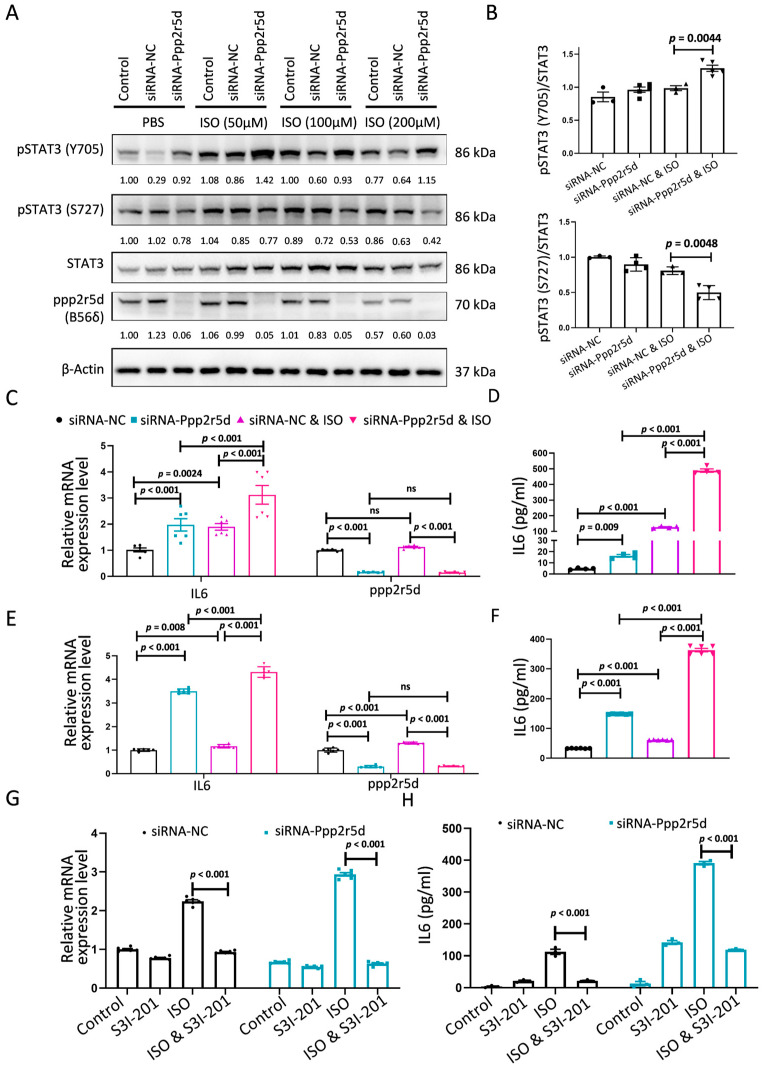
Knockdown *Ppp2r5d* in MCMs affected the status of STAT3 phosphorylation in the DCM model. (**A**) The expressions of the STAT3-related proteins were determined by Western blot, and representative images are shown. Total STAT3 and β-Actin were used as internal controls. The fold changes in protein expression level were normalized to the internal control and determined by densitometric analysis. Experiments were repeated three independent times. (**B**) The relative band intensity of pSTAT3 (Y705) and pSTAT3 (S727) was analyzed by ImageJ software (version 1.4.3.67) and normalized to Total STAT3. (**C**) Knockdown of *Ppp2r5d* resulted in upregulation of IL6 expression in mouse cardiomyocytes. Relative mRNA level of IL6 and *Ppp2r5d* in MCMs transfected with siRNA-NC or siRNA-*Ppp2r5d* after ISO treatment for 18 h, normalized to β-Actin expression (*n* = 6). (**D**) The protein level of IL6 in culture supernatants of MCMs among individual groups (*n* = 3). (**E**) Relative mRNA levels of IL6 and *Ppp2r5d* in NMCMs transfected with siRNA-NC or siRNA-*Ppp2r5d* after ISO treatment, normalized to β-Actin expression (*n* = 6). (**F**) The IL6 protein levels in culture supernatants of NMCMs were determined by an ELISA kit in each group (*n* = 3). (**G**) Relative IL6 mRNA level in MCMs transfected with siRNA-NC or siRNA-*Ppp2r5d* after ISO or S3I-201 treatment for 18 h. The values were normalized to β-Actin mRNA levels (*n* = 6). (**H**) The IL6 protein levels in culture supernatants of NMCMs were determined with an ELISA kit in each group (*n* = 3). Data are presented as the mean ± SD with *p* values indicated.

As a regulatory subunit of PP2A (serine/threonine protein phosphatase 2A), *Ppp2r5d* modulates signal transduction pathways by dephosphorylating specific target sites. Therefore, it is imperative to elucidate the mechanisms underlying the mitochondrial dysfunction induced by knocking down *Ppp2r5d* in the hypertrophy model. Previous research has demonstrated that STAT3 can be phosphorylated at two sites, namely serine residue 727 and tyrosine residue 705, resulting in changes to its intracellular localization and subsequent initiation of various biological processes [17,18]. Our findings demonstrate that inhibition of *Ppp2r5d* results in a distinct modulation of STAT3 phosphorylation levels, characterized by a decrease at Ser727 and an increase at Tyr705. This suggests that *Ppp2r5d* could mediate STAT3 modification to exert intricate modulation for the maintenance of mitochondrial function, potentially impacting ATP production or ROS level, both crucial for the metabolic function of cardiomyocytes (Figure 5A,B) [19]. For example, mtSTAT3 (pSTAT3 (S727)) interacts with GRIM-19, a component of the ETC complex I, subsequently enhancing the activity of complex I [20].

Phosphorylated STAT3 at Y705 forms dimers that translocate into the nucleus to mediate the transcription of downstream genes, including IL6 [21]. To investigate the potential role of *Ppp2r5d* in regulating IL6 through STAT3 modification, we evaluated IL6 expression in cardiomyocytes using RT-qPCR and ELISA. Knockdown of *Ppp2r5d* in cardiomyocytes stimulated with ISO significantly increased both IL6 mRNA expression and protein levels (Figure 5C,D). Consistently, the alteration was also confirmed in primary neonatal mouse cardiomyocytes (NMCMs) under the same conditions (Figure 5E,F). Furthermore, inhibition of Tyr705 phosphorylation of STAT3 with an inhibitor (S3I-201) reversed the upregulation of IL6 expression (Figure 5G,H) [22]. These findings provide evidence that *Ppp2r5d* modulates STAT3 Y705 phosphorylation to control IL6 levels in cardiomyocytes.

## 4. Discussion

In this study, we demonstrated that *Ppp2r5d* expression alteration in the DCM model resulted in a cascade of biological responses, including phosphorylation changes of mtSTAT3, decreased activity of the ETC complex, reduced ATP production, increased intracellular ROS levels, and exacerbated myocardial apoptosis. These changes further contribute to the abnormal DCM-related phenotype, such as elevated hypertrophic markers and IL6 levels, enlarged ventricles, impaired cardiac function, and accelerated ventricular remodeling. Additionally, exacerbated mitochondrial damage was observed in the DCM mouse model. These findings provide evidence for the significant role of *Ppp2r5d* in the pathogenesis of DCM.

Some factors such as inflammation, viral infection, and chemotherapeutic agents have been shown to contribute to the pathogenesis of DCM, ultimately leading to ventricular enlargement and HF. DCM manifests as dilated ventricles and impaired cardiac contractility in response to various causative stressors. Recently, we identified the alteration of *Ppp2r5d* mRNA in the peripheral plasma of patients with DCM [2]. This abnormal expression pattern has also been observed in the heart tissue of DCM mice, motivating us to further investigate the potential role of *Ppp2r5d* in the pathogenesis of DCM. It has been reported that the B56δ protein, encoded by *Ppp2r5d*, regulates numerous critical biological processes such as DNA replication and mitosis via distinct signal transduction pathways [23,24]. The primary form of PP2A is a heterodimeric trimeric complex consisting of a catalytic C subunit, a scaffold A subunit, and a regulatory B subunit. The regulatory B subunits comprise a large group of genetically and structurally diverse proteins that fall into four categories, B/B55, B′/B56, B′′/PR72, and B′′′/striatins. In mammals, B′/B56 contains five spliceosomes: B56α, B56β, B56γ B56δ, and B56ε. Previous studies on B56δ were focused on developmental disorder syndromes such as mental retardation, epilepsy, macrocephaly, and developmental delay [4,25]. *Ppp2r5d* knockdown promotes tumorigenesis and malignant development by enhancing phosphorylation at the Ser9 site of GSK-3β and the Ser62 site of the proto-oncogene C-myc [3]. The B56δ E420K mutation leads to an overactive AKT-mTOR signaling pathway via the regulation of AKT, GSK3α, and S6 hyperphosphorylation, resulting in an uncoordinated cell proliferation [24]. Inhibition of tumorigenesis can be achieved through the PP2A/GSK3β/MCL-1 pathway. Under hypoglycemia/metformin treatment, upregulated *Ppp2r5d* dephosphorylates GSK-3β, leading to a decline in MCL-1 and cell death [26]. ISO stimulation enhances PP2A activity by upregulating B56δ Ser573 phosphorylation. Mutation at the active site of B56δ can inhibit PP2A activity, leading to subsequently elevated intracellular phosphorylation of cTnI at Ser22/23 and of cMyBP-C at Ser282 in cardiomyocytes [7]. These findings suggest a potential function for *Ppp2r5d* in cardiomyopathy, although the mechanism remains unclear.

We observed that the *Ppp2r5d* mRNA levels were downregulated in the cardiac tissue of ISO-induced DCM mice, but not in MI mice. Surprisingly, our microarray results showed that *Ppp2r5d* knockdown of MCMs results in a highly enriched differential expression of genes involved in the pathways associated with hypertrophy and DCM. These data provide further evidence that *Ppp2r5d* is indeed associated with ISO-induced DCM.

Next, we found that *Ppp2r5d*-knockdown MCMs are more sensitive to the detrimental cellular effects of the H_2_O_2_-induced ROS. For example, intracellular ROS levels were significantly upregulated in the *Ppp2r5d*-deficient group compared to the control group. Indeed, oxidative stress induced by mitochondrial dysfunction is also involved in the pathogenesis of DCM [27]. Our results also demonstrated that *Ppp2r5d* deficiency contributes to a decrease in intracellular ATP levels in ISO-stimulated cells. We further revealed that the baseline mitochondrial respiration, ATP production, and maximal oxygen consumption were considerably reduced in the *Ppp2r5d*-deficient MCMs compared to control cells under stimulation. In the in vivo experiments, DCM mice that were treated with AAV-cTnT-shRNA-*Ppp2r5d* exhibited severe cardiac dysfunction, ventricular fibrosis, and ventricular dilatation compared to the control group. In addition, the ventricles of *Ppp2r5d*-deficient DCM mice showed significantly more myofilament and mitochondrial damage than did those of the vehicle-treated DCM mice. Notably, the expression levels of genes associated with ETC and ATP synthesis, such as *Ndufv1, Sdhb, Cyc1, Uqcrc2*, *and Atp5a1* were downregulated in the DCM model. The activity of the ETC complex I was also impaired in this condition.

STAT3 can be phosphorylated at two sites to modulate its biological function. Phosphorylation at site S727 drives STAT3 into the mitochondria (mtSTAT3) to participate in electron transport chain activity [28,29,30], while phosphorylation at site Y705 drives STAT3 into the nucleus, where it functions as a transcriptional activator and influences the pathophysiology of cardiomyopathy. For instance, inhibition of STAT3 Y705 phosphorylation was shown to significantly enhance cardiac function in a rat model of stress-induced cardiac hypertrophy [31]. Intriguingly, our research revealed that *Ppp2r5d* uniquely regulates the differential phosphorylation of STAT3 at these two sites. Knockdown of *Ppp2r5d* increased Tyr705 phosphorylation but suppressed Ser727 phosphorylation in the ISO-induced DCM model. The phosphorylation pattern of STAT3 leads to mitochondrial dysfunction and elevated IL6 levels in cardiomyocytes, thereby contributing to the development of interstitial fibrosis in DCM.

Collectively, our data suggest that *Ppp2r5d* is involved in the development of DCM and may represent a turning point in the elucidation of the pathogenesis of DCM. One possible mechanism is that *Ppp2r5d* regulates STAT3 phosphorylation to maintain mitochondrial energy homeostasis in normal myocardial tissue. During DCM development, downregulation of *Ppp2r5d* promotes mitochondrial dysfunction and apoptosis in cardiomyocytes as well as aggravating myocardial fibrosis and ventricular remodeling.

However, despite our identification of the *Ppp2r5d*-dependent axis that regulates the functional integrity of cardiomyocytes disrupted by DCM pathogenesis, open questions remain to be answered in the future. It remains unclear whether *Ppp2r5d* directly or indirectly modulates the phosphorylation of STAT3 at two specific sites. Previous research has suggested that *Ppp2r5d* may regulate the phosphorylation of S282 in cMyBP-C without affecting phosphorylation at S273 and S302, indicating potential differential regulation by different PP2A holoenzymes on distinct sites of the same protein [7]. Our findings indicate that knocking down *Ppp2r5d* upregulates phosphorylation at site Y705 and promotes the expression of IL6. It should also be noted that the phosphorylation of STAT3 (pSTAT3 (S727)) may be indirectly regulated by *Ppp2r5d* and requires further investigation. Furthermore, rescue experiments are necessary to restore normal levels in deficient *Ppp2r5d* cardiomyocytes and impede the progression of ISO-induced DCM.

In summary, we discovered that the knockdown of *Ppp2r5d* in ISO-induced cardiomyocytes increases the phosphorylation of STAT3 at the Y705 site, leading to an upregulation of IL6 level. Meanwhile, the phosphorylation of STAT3 at the S727 site decreases, which impairs the function of electron transport chain, resulting in massive ROS production. In vivo experiments further confirmed that *Ppp2r5d* can exacerbate ISO-induced ventricular dilation and HF. Overall, our findings reveal *Ppp2r5d* to be a crucial factor in the progression of DCM and provide a novel therapeutic target for the treatment of DCM.

## 5. Conclusions

This study has revealed, for the first time, the association between *Ppp2r5d* and Dilated cardiomyopathy. Our findings demonstrate that *Ppp2r5d*-deficient cardiomyocytes display elevated phosphorylation levels of STAT3 at Y705, resulting in an upregulation of IL6 expression. Conversely, the absence of *Ppp2r5d* reduces phosphorylation levels at S727 of STAT3, disrupting mitochondrial electron transport chain function and leading to dysregulation of ATP synthesis and ROS levels. Our research provides novel insights into the molecular mechanisms of *Ppp2r5d* in ISO-induced DCM, suggesting it as a potential therapeutic target for the treatment of this disease.

## Data Availability

The Microarray data have been deposited in the NCBI Gene Expression Omnibus under the accession number GEO: GSE238141. The original contributions presented in the study are included in the article, further inquiries can be directed to the corresponding author/s.

## Supplementary Materials

The following supporting information can be downloaded at https://www.mdpi.com/article/10.3390/biomedicines12081887/s1: Figure S1: *Ppp2r5d* inhibition promoted H_2_O_2-_induced apoptosis in MCMs.; Figure S2: *Ppp2r5d* inhibition promoted ISO-induced intracellular ROS in MCMs.; Figure S3: AAV-mediated knockdown of *Ppp2r5d* specifically in cardiomyocytes.; Table S1: List of primers for real-time PCR.
