# Supplementary material for: Unmasking Protein Phosphatase 2A Regulatory Subunit B as a Crucial Factor in the Progression of Dilated Cardiomyopathy
Source: Biomedicines. 2024 Aug 19;12(8):1887. doi: 10.3390/biomedicines12081887 (PMC11352103; doi:10.3390/biomedicines12081887)
Supplement: Supplementary file 1 [file biomedicines-12-01887-s001.zip › biomedicines-3135812-supplementary.pdf]

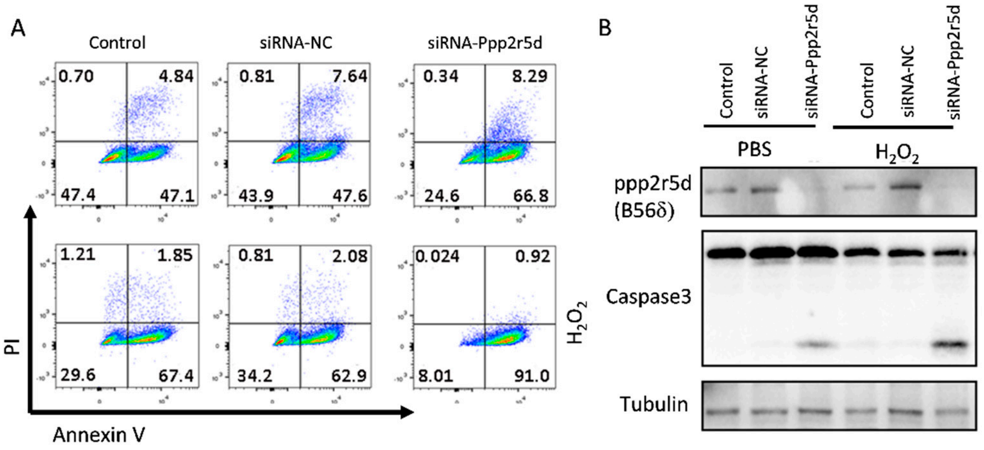


**Figure S1.** *Ppp2r5d* inhibition promoted $H_2O_2$ induced apoptosis in MCMs. **(A)** Flow cytometry with Annexin V-FITC/PI staining was performed to detect the apoptosis of each group with or without $H_2O_2$ treatment. Annexin V+/PI+: dead cells; Annexin V−/PI+: late-stage apoptosis cells; Annexin V+/PI−: early-stage apoptosis cells; Annexin V− PI−: live cells. **(B)** WB detected the expression of *Ppp2r5d* (B56δ) and Cleaved Caspase 3.

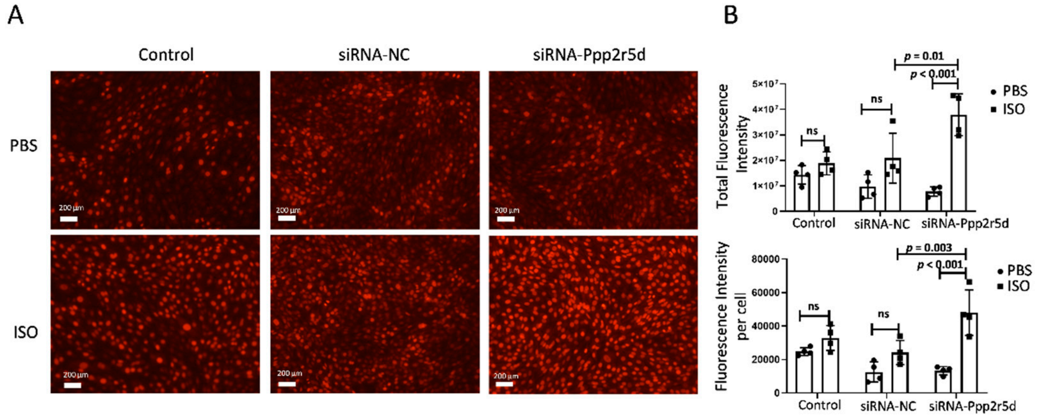


**Figure S2** *Ppp2r5d* inhibition promoted ISO induced intracellular ROS in MCMs. **(A)** Representative images of the in situ dihydroethidium (DHE) staining to monitor the superoxide generation in the indicated groups. Scale bar: 200 μM. **(B)** Quantification of fluorescence intensities and intensities per cell (Mean ± SD, *n* = 4).

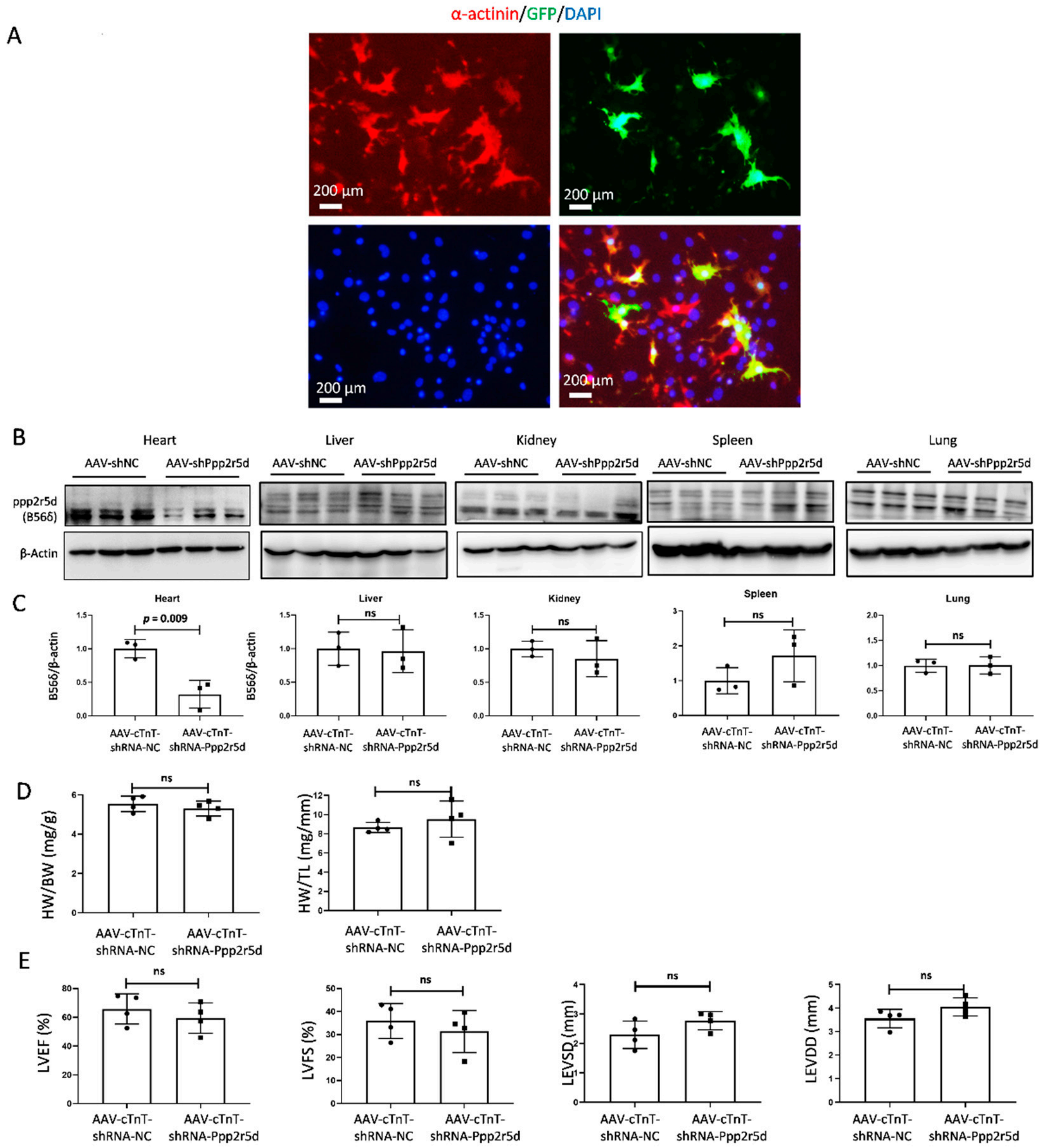


**Figure S3** AAV-mediated knockdown of *Ppp2r5d* specifically in cardiomyocytes. (A) Immunofluorescence staining was performed to evaluate the specific infection. The primary murine neonatal cells were isolated from heart tissue which contained not only cardiomyocytes but also fibroblasts. Representative immunofluorescent staining for *α*-actinin (red, cardiomyocytes), GFP (green, AAV-infected cells), and DAPI (blue, nucleus) in murine neonatal cells with AAV- cTnT -shRNA-*Ppp2r5d* infection. Scale bar: 200 μM. **(B)** Murine tissues were collected 5 weeks after AAV-cTnT-shRNA-*Ppp2r5d* and AAV-cTnT-shRNA-NC injections. Western blot was performed to evaluate the knockdown effect on *Ppp2r5d* in vivo. **(C)** The relative band intensity of Ppp2r5d (B56δ) was analyzed by ImageJ (version 1.4.3.67) and normalized to total β-Actin. *n*=3. Data are presented as Means ± SD with P values indicated. **(D)** Heart weight/body weight ratio and Heart weight/tibia length ratio in each group of mice. Data are presented as the mean ± SD with *p* values indicated. *n* = 4. (E) Quantitation of the left

ventricles' parameters in each group. LVEF: left ventricular ejection fraction; LVFS: left ventricular fraction shortening; LEVDD: left ventricular end-diastolic dimension; LEVSD: left ventricular end-systolic dimension. Data are presented as the mean ± SD with $p$ values indicated. $n = 4$.

Table S1. List of primers for real time PCR.

| Gene | Forward (5'-3') | Reverse (5'-3') |
|---|---|---|
| Mouse Nppa | TACAGTGCGGTGTCCAACACAG | TGCTTCCTCAGTCTGCTCACTC |
| Mouse Nppb | TCCTAGCCAGTCTCCAGAGCAA | GGTCCTTCAAGAGCTGTCTCTG |
| Mouse IL6 | TACCACTTCACAAGTCGGAGGC | CTGCAAGTGCATCATCGTTGTTC |
| Mouse Myh7 | GCTGGAAGATGAGTGCTCAGAG | TCCAAACCAGCCATCTCCTCTG |
| Mouse Ppp2r5d | GAACACAAGGTGTTTCTCGTCCG | TCCTTCTCCAGGAACTGCACCA |
| Mouse β-actin | CATTGCTGACAGGATGCAGAAGG | TGCTGGAAGGTGGACAGTGAGG |
| Mouse Nd1 | TCCGAGCATCTTATCCACGC | GTATGGTGGTACTCCCGCTG |
| Mouse Ndufv1 | GGTATCTGTGCGTTTCAGCA | GATCCGGTCTTCATCCTTCA |
| Mouse Sdhb | ACTGGTGGAACGGAGACAAG | GCAGCGGTAGACAGAGAAGG |
| Mouse Cyc1 | ACCTGGTGGGAGTGTGCTAC | CATCATCATTAGGGCCATCC |
| Mouse Uqcrc2 | AATGGCTCTGGTTGGACTTG | ACGGTATTTGGCTTTTGCAC |
| Mouse Co1 | CTACCCACCTCTAGCCGGAA | TGTTATGGCTGGGGGTTTCA |
| Mouse Co2 | ACCGAGTCGTTCTGCCAATA | ATTTAGTCGGCCTGGGATGG |
| Mouse Atp5a1 | GCCCTCGGTAATGCTATTGA | CACAGAGATTCGGGGGATAA |
| Mouse ATP6 | ACGCCTAATCAACAACCGTC | TTCGTCCTTTTGGTGTGTGGA |
| Mouse Vdac1 | AGTGACCCAGAGCAACTTCGCA | CAGGCGAGATTGACAGCAGTCT |
